# Preventing Smoking in Young People: A Systematic Review of the Impact of Access Interventions

**DOI:** 10.3390/ijerph6041485

**Published:** 2009-04-20

**Authors:** Lindsay Richardson, Natalie Hemsing, Lorraine Greaves, Sunaina Assanand, Patrice Allen, Lucy McCullough, Linda Bauld, Karin Humphries, Amanda Amos

**Affiliations:** 1 British Columbia Centre of Excellence for Women’s Health; E311, 4500 Oak Street, Box 48, Vancouver, BC V6H 3N1, Canada; E-Mails: linds321@hotmail.com (L.R.); lgreaves@cw.bc.ca (L.G.); sassanand@cw.bc.ca (S.A.); pallen@cw.bc.ca (P.A.); 2 Department of Educational and Counselling Psychology, and Special Education Counselling Psychology Doctoral Program, University of British Columbia, 2125 Main Mall, Vancouver, BC V6T 1Z4, Canada; E-Mail: lmccullough@cw.bc.ca; 3 Department of Social and Policy Sciences and UK Centre for Tobacco Control Studies, University of Bath, Bath, BA2 7AY, U.K.; E-Mail: L.Bauld@bath.ac.uk; 4 Cardiac Registry, Provincial Health Services Authority, 700-1380 Burrard St. Vancouver, BC, V6Z 2H3, Canada; E-Mail: khumphries2@phsa.ca; 5 Public Health Science Section, Division of Community Health Sciences, The University of Edinburgh, Teviot Place, Edinburgh EH8 9AG, U.K.; E-Mail: amanda.amos@ed.ac.uk

**Keywords:** Access restrictions, illegal sales, tobacco, youth, prevention

## Abstract

**Aims:**

To examine existing evidence on the effectiveness of interventions that are designed to prevent the illegal sale of tobacco to young people. The review considers specific sub-questions related to the factors that might influence effectiveness, any differential effects for different sub-populations of youth, and barriers and facilitators to implementation.

**Methods:**

A review of studies on the impact of interventions on young people under the age of 18 was conducted. It included interventions that were designed to prevent the illegal sale of tobacco to children and young people. The review was conducted in July 2007, and included 20 papers on access restriction studies. The quality of the papers was assessed and the relevant data was extracted.

**Results:**

The evidence obtained from the review indicates that access restriction interventions may produce significant reductions in the rate of illegal tobacco sales to youth. However, lack of enforcement and the ability of youth to acquire cigarettes from social sources may undermine the effectiveness of these interventions.

**Conclusions:**

When access interventions are applied in a comprehensive manner, they can affect young people’s access to tobacco. However, further research is required to examine the effects of access restriction interventions on young people’s smoking behaviour.

## 1. Introduction: Preventing Tobacco Use among Youth

Smoking among young people is of concern due to the addictive nature of tobacco and the health risks associated with tobacco use. A focus on the prevention of the uptake of cigarette smoking in youth is of particular importance as the majority of smokers initiate smoking or become habitual smokers prior to the age of 18 [1,2] and are less likely to give up smoking than those who start later in life [3]. The prevalence of smoking among young people is affected by sex and gender and reflects diversity and inequality. Risk factors associated with youth smoking include low socioeconomic status, being female, mental illness, low parental education and living in a single parent household [3]. In addition to these socio-demographic factors, youth smoking behavior is also influenced by peer pressure and exposure to positive images of smoking in the media.

Despite the fact that youth smoking rates have declined over the past two decades in the UK, regular smoking in young people remains a public health issue. In England, the prevalence of regular smoking among young people aged 11 to 15 is 9% [4]. In the US, 6.8% of middle school students (grades 6–8, or ages 11–14) were current smokers in 2006 [5]. In Canada, the rate of current smokers in 2004–2005 among youth in grades 5 (age 10–11) to 9 (age 14–15) was 1.7% [6]. Furthermore, statistics indicate that smoking rates for girls are greater than, or equal to smoking rates for boys. Girls between the ages of 11 and 15 (10%) in the UK are more likely to be regular smokers than boys (7%) [4]. In the United States, smoking rates between middle school boys (6.3%) and girls (6.4%) are similar [7]. Analogouly, in Canada, smoking rates between boys (1.5%) and girls (1.8%) aged 10–15 are similar [6]. Regular smoking also increases with age. In England, 20% of 15 year olds are regular smokers compared to only 1% of 11 year olds [4]. Similarly, in Canada, 10.4% of boys and girls aged 15–17 are current smokers, compared to 1.7 % among 10–15 year olds [6]. In the US, smoking rates among high school students (grades 10–12, or ages 15–18) are much higher (19.4%), compared to middle school students (6.8%) [5]. In the short-term, young smokers are more likely to develop respiratory illness and face co-morbidity issues [3]. In the long term, youth who become regular smokers and continue smoking in adulthood are more likely to develop cancer and cardiovascular disease [2]. Therefore, it is essential to prevent cigarette use in young people.

Restricting young people’s access to cigarettes and tobacco has been a key component of tobacco legislation aimed at preventing the uptake of smoking. Therefore, the purpose of this paper is to examine the existing evidence on the effectiveness of interventions that are designed to prevent the illegal sale of tobacco to young people.

## 2. Methods

In this review, interventions designed to prevent the illegal sale of tobacco to young people included: (a) efforts to educate merchants and the general public about the minimum age law, (b) proof of age schemes (age or identification requests), and (c) regulation and law enforcement (including encouraging members of the community to help enforce the law).

### 2.1. Literature Searches

The literature searches were conducted in July 2007 and covered studies published between 1990 and 2007 in the following standard databases: ASSIA (Applied Social Sciences Index and Abstracts), BNI (British Nursing Index), CDSR, CENTRAL, CINAHL, Current Contents, DARE, EMBASE, HMIC, HSTAT, MEDLINE, National Research Register, PAIS, PsycINFO, SIGLE (System for Information on Grey Literature in Europe Archive), Social Policy and Practice, Sociological Abstracts, and TRIP (Turning Research Into Practice). A total of 7,365 mass media and access restriction titles and abstracts were screened, from which 184 papers were selected for further review.

Full copies of these studies were obtained and were independently assessed for inclusion by two reviewers. Of these studies, 60 (40 mass media, 20 access restriction) met the inclusion criteria for this rapid review, 45 studies (34 access restriction; 11 mass media) were excluded from the review, and the remaining 79 studies were incorporated as background material. In order to address the research questions, studies were analyzed for any relevant primary or secondary data, which was then extracted and included in the review. Studies that did not directly relate to the review, describe an intervention, or address the research questions or outcomes of interest were excluded. The access restrictions literature forms the basis of this review.

Additionally, individual studies reviewed by the Cochrane Reviews and other systematic reviews, and narrative reviews were not included or extracted in this review. The included literature reviews have been used as a key source of evidence, rather than attempting to summarise all of the individual studies identified (this also prevented reporting studies more than once). It is also important to note that studies identified by the included systematic and narrative reviews were based on different eligibility criteria and outcomes of interest. A list of excluded access restriction studies (n=34) with reasons for exclusion is presented in Appendix B.

This review, although international, excludes studies published in languages other than English and studies conducted in developing countries. Inclusion criteria include studies that examine the impact of interventions on young people under the age of 18 and studies that examine interventions designed to prevent the illegal sale of tobacco to young people under the age of 18.

### 2.2. Rating the Evidence

The strength of the evidence was determined using a model developed by the National Institute for Health and Clinical Excellence (NICE), an internationally respected government organization responsible for providing guidance on promoting good health and preventing and treating ill health in the United Kingdom. All of the studies that met the inclusion criteria were rated by two independent reviewers in order to determine the strength of the evidence. Once the research design of each study was determined (using the NICE algorithm), studies were assessed for their methodological rigour and quality based on the critical appraisal checklists provided in Appendix B of the *Public Health Guidance Methods Manual* [8] (see Table 1; appraisal checklists examine a variety of factors specific to each study design including reliability, validity, confounders, randomisation, concealment, missing data, and eligibility. For more information regarding appraisal checklists please refer to the *Public Health Guidance Methods Manual).* Each study was categorised by study type and graded using a code ‘++’, ‘+’ or ‘–’, based on the extent to which the potential sources of bias had been minimised. Inter-rater reliability was employed, such that those studies that received discrepant ratings from the two reviewers were resolved by consulting a third reviewer. Following the rating process, a narrative synthesis of key results was developed. It was not possible to conduct a meta-analysis as the studies included in the review were heterogeneous in design and the type and range of outcomes varied significantly between studies.

### 2.3. Summary of Findings

The key question of this literature review was:

1. Which interventions are effective in reducing the illegal sale of tobacco to children and young people?

There are nine sub-questions that are addressed:

- What impact do access restrictions have on youth smoking behaviour and stage of smoking?
- When interventions can be compared, which are most effective in reducing illegal tobacco sales to youth?
- Are the interventions delaying rather than preventing the onset of smoking?
- How does the way that the intervention is delivered influence effectiveness?
- Does effectiveness depend on the status of the merchant?
- Does the site/setting influence effectiveness?
- Is sustained implementation or enforcement important?
- How does effectiveness vary according to the age, sex or ethnicity of young people?
- What are the facilitators and barriers to implementation?

## 3. Results

### 3.1. What Impact Do Access Restrictions Have on Youth’s Smoking Behaviour and Stage of Smoking?

Nearly all of the studies looked at the effect of interventions on illegal sales rather than individual smoking behaviour or prevention of uptake. One exception is a 2002 systematic review (+) by Fichtenburg and Glantz which addressed the impact of access restrictions on smoking prevalence, but found no difference in youth smoking in communities with youth access interventions and control communities [9]. The pooled estimate of the mean difference in 30-day prevalence was −1.5% (95% confidence interval; −6.0% to +2.9%). Interventions in their review included: simple enforcement of minimum age restrictions, retailer and community education of minimum age laws, and education combined with active enforcement via compliance testing of vendors, warnings, fines and suspension of tobacco selling licenses. Furthermore, all four controlled studies included in the review reported merchant compliance with minimum age restrictions (i.e. asking for identification and not selling to persons underage) of 82% or higher, yet failed to demonstrate decreased smoking by young people.

Ross and colleagues examined in 2006 the differential effects of cigarette prices, clean indoor air laws, and youth access laws on smoking uptake among US high school students (cross-sectional, +) [10]. They found that merchant compliance with youth access laws reduced the probability of youth being in higher stages of smoking uptake (p<0.05). Moreover, they found that the impact of compliance was greater for those who were in later stages of uptake; at earlier stages of smoking uptake, cigarettes may be more often obtained from friends and other social sources.

Given that only two studies addressed the impact of interventions on smoking behaviour, it is not possible to draw definitive conclusions. Nevertheless, the studies were both rated positively (+), and indicate that access restrictions may have little impact on young people’s smoking behaviour and that the impact of access restrictions on smoking behaviour may depend upon stage of smoking uptake.

### 3.2. When Interventions Can Be Compared, Which Are Most Effective in Reducing Illegal Tobacco Sales to Youth?

#### 1) Tobacco industry interventions

One cross sectional (−) study found that tobacco industry interventions are not effective in reducing illegal sales [11]. The authors examined the effectiveness of the Tobacco Institute’s “It’s the Law” campaign seven months after its launch in the US. They found that six of the seven participating merchants (86%) and 131 of the 149 non-participating merchants (88%) were willing to illegally sell cigarettes to young people. Yet, the study did not receive a positive rating (−), and therefore the results must be interpreted with caution. Specifically, there was a lack of information on sampling method, eligibility criteria, and the type of analysis conducted, and no p-values were provided. Further research is required to determine whether or not this particular tobacco industry-sponsored intervention was effective in reducing illegal sales.

#### 2) Multi-component interventions and active enforcement

Multiple studies suggest that interventions are most effective when they are multi-component (e.g. youth access policies, community and merchant education, vending machine policies), but that this can be undermined by weak enforcement of tobacco laws. For example, in an Australian study, Tutt and coworkers (cross-sectional, -) explored retail compliance with prohibition of sales to minors [12]. Findings revealed that compliance rates increased as a result of publicised prosecutions and initiation of a campaign aimed at increasing merchant awareness of minimum age legislation. Non-compliance rates were 30.8% in December 1994, 8.1% in May 1996 and 0% in 1998/9. However, this study did not receive a positive review (−) because confounders were not adequately accounted for, and therefore the results may not be conclusive.

Yet, one (+) review did find multi-component strategies to be successful in decreasing illegal sales to youth. Levy and Friend found that successful US-based policies that reduced retail sales usually had a multi-component approach, including severe enforcement and penalties, as well as community education and mobilisation [13]. Two studies in this review [11,14] revealed that merchant education and community and media campaigns were ineffective on their own in reducing tobacco sales. For example, although some stores may stop selling to youth because of youth access policies, other stores may increase their sales particularly if merchants are unlikely to be penalized, or if the community lacks concern. The review also found that vending machine policies that involved community and merchant education without locking devices or total vending machine bans had limited effect on sales to young people.

A Cochrane review by Stead and Lancaster (++) examined how interventions aimed at preventing illegal sales of tobacco can reduce underage access [15]. Although none of the strategies achieved 100% merchant compliance, the authors concluded that actively enforcing laws or using multi-component educational strategies was more effective than providing merchants with information about illegal sales. Finally, a New Zealand based cross-sectional study (−) evaluated the effectiveness of the Smoke-free Environment Act of 1990, prohibiting the sale of tobacco products to minors [16]. The study evaluated a nationwide programme of controlled purchase operations (CPOs) using volunteers under the age of 16. Controlled purchase operations describe attempts by youth/minors to purchase tobacco products, within the context of a study examining illegal tobacco sales. Between September (1996) and June (1997), 9.7% of CPOs resulted in illegal sales of tobacco, while 5.9% occurred between July and December (1997). By December (1997), 84% of the violating merchants were convicted. However, this study did not receive a positive rating, as no information was provided on the type of analysis conducted and the sampling frame used. Therefore there is only limited evidence from a single (++) review to suggest that active legal enforcement is useful for decreasing illegal sales of tobacco to minors and improves the success of multi-component strategies [15] .

#### 3) Age and Identification Requests

Results from three positively rated study (one ++, and two +) indicate that age, and even more so identification requests are useful in reducing illegal sales of tobacco to youth. In a US-based cross-sectional study (++) by Glanz and coworkers, only two variables were associated with whether a successful purchase attempt was made: whether minors’ age was requested (OR = 0.030, 95% CI = 0.002, 0.426) and whether minors’ identification was requested (OR = 0.001, 95% CI = 0.001, 0.020) [17]. These findings indicate that age and/or identification requests may be an effective means by which to decrease youth access to tobacco products. However, some evidence suggests a greater reduction in sales to minors when identification (ID) is requested than when age is requested. For example, an American study by Landrine and colleagues (+) found that, across 2,567 attempts to purchase, minors were asked their age 13.1% of the time and were asked to produce ID 4.1% of the time [18]. Yet, when ID was requested, minors were refused cigarettes 99% of the time and sales were less likely (χ^2^ = 16.8 p≤0.001). Consistent with these findings, a US cross-sectional study (+) conducted by DiFranza *et al.* found that sales occurred in 1.5% of 1,180 attempts when proof of age was requested, as compared to 64% of 712 attempts when it was not (p<0.001) [19]. In contrast, sales occurred in 5% of 317 attempts when age was asked, as compared to 30% of 1,502 attempts when it was not (p<0.001).

Nonetheless, results from a positively reviewed study revealed that young people who present identification may succeed in purchasing tobacco. In a non-randomised controlled trial (+), Levinson and coworkers examined the effect on cigarette sales when minors presented ID, compared with minors who did not present ID [20]. Sixteen minors conducted supervised tobacco purchase attempts in six urban and suburban communities in the US. Findings revealed that when clerks requested ID, sales were six times more frequent among minors who presented valid ID (identifying them as minors) than minors who did not present identification (12.2% vs. 2.0%, RR = 6.2, p<0.0001).

### 3.3. Does Effectiveness Depend on the Status of the Merchant?

In their 1996 US-based cross-sectional study (+) exploring the tobacco industry-sponsored “It’s the Law” compliance program, DiFranza and colleagues concluded that merchant participants and non-participants of the compliance program were just as likely to make illegal sales to minors (OR = 0.87, 95% CI = 0.59, 1.35, p = 0.0001) [21]. “It’s the Law” was an informational campaign aimed at tobacco retailers, which included information leaflets, and stickers to be displayed on retail counters advising of the minimum age law.

However, four positively reviewed (+) studies of other non-industry sponsored interventions suggest that illegal tobacco sales are impacted by the age, gender and ethnicity of the clerk. In a US non-randomised controlled trial (+), Levinson and coworkers found that during supervised purchase attempts, clerks perceived to be younger than 30 years of age were significantly more likely to sell tobacco to youth (9.9% of clerks under 30 made sales vs. 5.5% of clerks between 30–50 and 6.9% of clerks over 50) [20].

In a cross-sectional US-based study [19] (+) by DiFranza *et al.* illegal sales were more common when the clerk was male as opposed to female (27% vs. 22%; p<0.05). In a cross-sectional study (+) by Landrine and colleagues findings revealed that the gender of the clerk did play a role in identification request (p = 0.05) but not in asking minors their age (p = 0.07) [18]. Female clerks (32.4% of the time) were slightly more likely than male clerks (26.3% of the time) to ask children their age.

Landrine and co-workers’ (cross-sectional +) US-based study also found that the clerk’s ethnicity was associated with age requests (χ^2^ (4) = 19.60, p<0.001) [18]. For example, Asian clerks requested age more often (35.5%) than other ethnic groups: African American clerks (22.7%); Middle Eastern clerks (21.7%); White clerks (17.5%); and Latinos (8.5%). Ethnicity also played a role in requesting ID (χ^2^ (4) = 20.45, p<0.001). White clerks asked for ID 18.5% of the time, Latinos asked 15% of the time, Asians asked 7.5% of the time, Middle Eastern clerks asked 6.6% of the time and African Americans asked 2.3% of the time.

### 3.4. Does the Site/Setting Influence Effectiveness?

Evidence from four positively reviewed studies (three +, one ++) shows that site/setting does influence effectiveness of access restriction measures. In a Swedish cross-sectional study (+), Sundh and co-workers compared the ability of young people to purchase tobacco before and after the implementation of the minimum age requirement of 18 years in 1997 [22]. While most of the purchase attempts continued to occur in department and grocery stores, the results of purchase attempts in various settings differed before and after the implementation of the minimum age restriction. In 1999, 66% of purchase attempts in department and grocery stores were successful, compared to 84% in 1996 (p<0.001). In 1999, 78% of purchase attempts at newsstands and in tobacco shops were successful, compared to 96% in 1996 (p<0.001). Lastly, in 1999, 63% of adolescents successfully purchased tobacco in service stations, compared to 94% in 1996 (p<0.001). Glanz and colleagues carried out a cross-sectional study (++) in the US between 1996–2003, in which they examined minors aged 14–17 years who attempted to purchase tobacco products [17]. They found that 25.5% of purchases occurred in food stores, 44.7% occurred in convenience stores, 16.8% occurred in gas stations, and 13% occurred in other stores.

In particular, the presence of self-service displays and unlocked vending machines may increase young people’s ability to access tobacco products. In a cross-sectional US-based study [19] (+), DiFranza and colleagues found that illegal sales were comparable for locked vending machines (19% of 47 attempts) and over-the-counter outlets (24% of 1075 attempts; p>0.05), but were more frequent for self-service displays (37% of 75 attempts, p = 0.01 vs. over the counter) and unlocked vending machines (64% of 58 attempts, p<0.0001 vs. over the counter). Locked vending machines, or lockout devices, describe vending machines which require an employee to unlock a vending machine selling cigarettes, at the request of a customer. In a cross-sectional study (+), DiFranza and colleagues found that in communities without requirements for lockout devices, illegal sales were far more likely from vending machines than over-the-counter sources (OR = 3.0, 95% CI = 1.9, 4.7, p = 0.0001) [20].

### 3.5. Is Sustained Implementation or Enforcement Important?

Some positively rated evidence (from three + studies) suggests that in order for access restrictions to be effective, ongoing implementation is required. For example, in a 2006 US cross-sectional study (+), Sundh and Hagquist examined associations among merchant inspections (i.e. through test purchases), merchant compliance and access to tobacco by youth between 2001 and 2003 [23]. The researchers found that 32.3% of the 3980 first-time inspections resulted in violations for selling tobacco to a minor. In contrast, 25.9% of the second-time inspections of the same retailers resulted in violations for selling tobacco to a minor (p<0.05).

The implementation and sustained enforcement of minimum age laws among merchants may enhance tobacco-use prevention efforts for youth. In a 2006 Swedish cross-sectional study (+), Sundh and coworkers assessed three test locations in order to investigate regional differences in tobacco access and to inform authorities’ efforts to enforce compliance with minimum-age restrictions [24]. In 1996, 84% (n = 214) of test purchases in shops with a voluntary age-limit resulted in successful purchases. In contrast, in 2005, 48% (n = 900) of test-purchases were successful (p<0.001). The authors concluded that opportunities to purchase cigarettes were reduced by the introduction of a minimum-age law in 1997 that was supported by both merchants and the community. Together, these findings suggest that sustainability is a key issue to the effectiveness of access restrictions in preventing illegal tobacco sales to youth.

Finally, in a US-based cross-sectional study (+), Chaloupka and Grossman examined the effectiveness of various tobacco control policies, including: increased taxes, restrictions on smoking in public spaces and worksites, and limits on the availability of tobacco for youth [25]. The authors note that limited enforcement of these broad policies impedes the reduction of youth smoking. In particular, they argue that age restrictions are not well enforced, and are ineffective unless coupled with educational programs, licensing and fines.

### 3.6. How Does Effectiveness Vary According to the Age, Sex or Ethnicity of Young People?

#### 1) Age and Smoking Status

Some interventions may be more effective in reducing tobacco access and use by younger smokers, as highlighted in three positively reviewed studies (two +, one ++). In a US-based cross-sectional study (++), Glanz and colleagues found a decrease in youth tobacco purchases between 1996 (44.5%) and 2003 (6.2%); older youth were more successful in purchasing tobacco than their younger counterparts in 2003 (age 15: 0%, age 16: 4.7% and age 17: 9.2%), however this difference was not significant (p>0.05) [17]. Consistent with this finding, in an American cross-sectional study (+), DiFranza and colleagues found that merchants were more likely to sell tobacco products to older youth; violation rates varied from 4% for youth aged 13 years, to 30 % for youth aged 16 years (p<0.01) [19]. In a US non-randomised controlled trial (+), Levinson and coworkers (2002) found that minors who were aged 17 had significantly greater odds of purchasing cigarettes than minors (p<0.01) [20].

Age of appearance may also influence minors’ ability to access tobacco products, according to findings from two (+) positively rated studies. In Swedish trials carried out in 1999 and published in 2004, Sundh and co-workers (+) found that 72% of attempted purchases by younger looking adolescents were successful, whereas 92% of attempted purchases by older looking adolescents were successful [22]. Similarly, in a US cross-sectional study (+), Levinson and colleagues concluded that minors who appeared to be 16–17 years old were more successful in purchasing tobacco than minors who appeared to be 11–15 years old (OR = 3.4, 95% CI = 2.0, 5.8, p< 0.0001) [21].

One study also examined whether access restrictions were more effective in reducing tobacco access and use by lighter versus heavier smokers. In an Australian cross-sectional study (−), Tutt and coworkers found that after three years of 90% retail compliance, smoking rates for youth aged 12–17 years decreased from 25.9% in 1993 to 22.7% in 1996, and to 17.1% in 1999 [12]. The greatest reduction could be found among persons who smoked 1 to 5 cigarettes a day, however this finding was not statistically significant (χ^2^ = 18.4, p = 0.182). Furthermore, confounders were not accounted for in this study, and therefore this study was not positively reviewed. Therefore, further research is required to examine differences in the effect of minimum age restrictions for lighter and heavier smokers.

#### 2) Sex

Three positively rates studies (one ++, two+) indicate that girls and boys differ in their ability to successfully purchase tobacco products. In a US cross-sectional study (+), DiFranza and colleagues found that girls had greater purchase success rates than boys (OR = 1.49, 95% CI; 1.01, 2.19, p < 0.05) [21]. In contrast, other research has found that boys are more successful in purchasing tobacco than girls. In a Swedish cross-sectional study (+), Sundh and co-workers examined the impact of the introduction of a minimum age law in 1997 on tobacco purchases by youth. For girls, they found that 84% of purchase attempts in 1996 and 65% of purchase attempts in 1999 were successful (p<0.001) [22]. For boys, they found that 96% of purchase attempts in 1996 and 85% of purchase attempts in 1999 were successful (p<0.001). Glanz and colleagues (++) found that tobacco purchases decreased from 1996 (44.5%) to 2003 (6.2%), yet more sales occurred for boys (9.3%) than girls (4.5%), although this difference was not statistically significant (p>0.05) [17].

Further, the implementation of minimum age restrictions may impact girls and boys differently, according to findings from one (+) study. In a cross-sectional study, Sundh and colleagues analysed adolescent’s access to tobacco before and after the introduction of a minimum age law in Sweden [26]. Findings revealed that the proportion of boys and girls in year 7 of school who said that they had bought tobacco during the previous month had decreased significantly from 11.5% to 7.8% and from 11.6% to 6.9%, respectively (both p<0.0001). For smokers, the proportion of girls who bought tobacco in shops decreased (p<0.001) in all age groups (year 7: 93.8% to 74.1%; year 9: 94.3% to 84.8%; year 2 of upper secondary school: 96.4% to 90.7%). Corresponding figures for boys who smoked showed a statistically significant decrease only among year 9 students (92.8% to 87.6%, p<0.05).

#### 3) Ethnicity

Young people of different ethnicities may vary in their ability to purchase cigarettes, according to findings from one positively reviewed (+) study. Landrine and colleagues (+) found that African American youth (5.3%) were significantly more likely than White youth (3.1%) χ^2^ (1) = 4.65, p = 0.03), but not more likely than Latino youth (4.4%, χ^2^ (1) = 1.72, p = 0.19) to be asked for ID [18]. When African American youth were asked for ID, sales were refused 100% of the time, as opposed to 79.2% of the time when ID was not requested (χ^2^(1) = 9.56, p = 0.002). However, it must be noted that these findings are specific to the American context, and likely cannot be generalized to other countries.

### 3.7. What Are the Facilitators and Barriers to Implementation?

Four positively rated reviews (one ++, three +) indicate that social sources limit the effectiveness of minimum age restrictions in reducing youth’s ability to procure cigarettes. According to a review (++) by Lantz and colleagues, one of the major barriers to the effective implementation of youth access restrictions is the ability for youths to acquire tobacco through social sources, such as family members, friends and strangers [27]. Consistent with this assertion, two (+) reviews [9,28] by Fichtenberg and Glantz [9] and Backinger and colleagues [28] note that social sources of cigarettes act as a barrier to the effective implementation of access laws. As young people find it harder to buy cigarettes from commercial sources, they tend to shift to other available resources. In their review, Levy and Friend (+) suggest that research should consider non-retail sources of tobacco such as parents, older siblings, peers and black markets [13].

## 4. Discussion

### 4.1. Limitations

There are a number of limitations to this review. While the literature search was international in scope, the majority of the articles identified within this review referred to US specific interventions or laws/restrictions. Since the demographics of participants in US studies differ to the demographics of young people in other countries, it is not clear whether all findings are applicable to youth in a variety of global contexts. Yet some general lessons, such as the usefulness of comprehensive tobacco control interventions, will likely be applicable to a variety of contexts.

A second limitation of this review is that many of the studies identified used very similar study designs. Most of the studies identified by the literature search were observational in nature. Only one study [20] was experimental; the majority used a cross-sectional research design. Many of these studies relied on recall or self report data. Because the data were predominantly based on self-reports, it could be argued that adolescent’s reports of purchase attempts may be subject to recall bias. However, studies have shown that in regards to their own smoking behaviour, children's and adolescent's reports are consistent over time [29,30]. Therefore, self reports may be informative, but could be enhanced with actual observations of the purchase attempt.

### 4.2. Conclusions

Findings from this review suggest that when access interventions are implemented in a comprehensive manner, they can decrease the illegal sale of tobacco to young people. Interventions that are multi-component in nature and with active and ongoing enforcement are the most successful. Specifically, findings revealed that combined, successive retail inspections, public prosecutions and awareness of minimum age restrictions decrease illegal sales of tobacco.

Although one review (+) [9] found no differences in smoking rates in communities with and without access restrictions, there is a body of evidence indicating that the way an intervention is implemented impacts effectiveness. A variety of factors can influence the effectiveness of access restrictions, such as whether clerks ask potential buyers to confirm age or identification, the person (e.g. sex, ethnicity of the clerk) who is delivering the intervention, and the site/setting of the intervention. For example, store clerks who are younger and male may be more likely to sell tobacco to youth. Therefore, interventions that train or educate merchants may be more effective if they are tailored according to the age and/ or gender of the merchant. The effectiveness of an intervention can also be influenced by age, sex, diversity and stage of smoking of the potential buyer, suggesting that complementary tailored youth focused intervention strategies (education, mass media campaigns, etc.) are required.

Finally, there are various factors which may impede the effectiveness of access restrictions in preventing smoking among youth. Nearly all of the studies identified by the literature search examined the effect of interventions on illegal sales (e.g. number of sales to youth, merchant compliance) rather than behaviour. One study did examine the impact of access restrictions on smoking behaviours and found no relationship between merchant compliance and smoking prevalence [9]. As a result, it is not clear what impact access restrictions are having on smoking behaviours. However, some evidence suggests that youth in the early stages of smoking may not be impacted as much by access restrictions due to alternative sources of tobacco. While age, and even more so, identification request can decrease the illegal sale of tobacco to youth, youth may also acquire cigarettes through social sources. Youth may also be able to buy cigarettes singly (although this is illegal irrespective of age) or in packs of ten which make cigarettes more affordable. Furthermore, despite the fact that illegal sales to youth continue, very few store clerks have been prosecuted by the law or given any fines [5]. Lack of enforcement is a key barrier to reducing the illegal sale of tobacco to youth. Yet, youth can also access cigarettes through the internet and vending machines and may also have access to contraband cigarettes (unlawful or illegally traded cigarettes, such as generic cigarettes). Further research is required to examine these processes, as well as the impact that access restrictions have on the smoking behaviour of young people. However, general lessons such as the usefulness of comprehensive interventions and the strict enforcement of minimum age restrictions are generally applicable in reducing the illegal sale of tobacco to youth.

## Figures and Tables

**Table 1.** Type and quality of evidence.

**Type and quality of evidence**	
Randomised Control Trial (RCT)	
Meta Analyses	
Systematic Reviews	
Case Control Studies	
Cohort Studies	
Controlled Before and After (CBA) Studies	
Interrupted Time Series (ITS) Studies	
Qualitative Studies	
Cross-sectional Studies	
**Grading the evidence**	
++	**All or most of the quality criteria have been fulfilled** Where they have been fulfilled the conclusions of the study or review are thought *very unlikely* to alter
+	**Some of the criteria have been fulfilled** Where they have been fulfilled the conclusions of the study or review are thought *unlikely* to alter
−	**Few or no criteria fulfilled** The conclusions of the study are thought *likely or very likely* to alter

**Table 2.** Access Restriction Evidence Table.

**First author** **YearCountry** **Study design** **Quality**	**Study population** **Inclusion/exclusion criteria.** **Number of participants (randomised to each group or otherwise).** **Age; Sex; S/E status; Ethnicity; Pregnant; Other, e.g. inpatient, ….**	**Research question** **Power calculation** **Funding**	**Intervention** **Comparisons** **Length of follow-up, follow-up rate**	**Main results** **Effect size** **CI**	**Confounders** **Comments**
Backinger *et al.* 2003 USA Review (narrative synthesis) +	Data included smoking prevention studies published from January 1990 to May 2002 and conducted in the US. All identified smoking cessation studies for adolescents. Young adult data was limited to initiation and cessation studies.	To summarize the evidence on adolescent and young adult prevention and cessation, and provide future directions for research. Funder not mentioned.	Data was collected from published literature. Pubmed, PsychInfo, ERIC and SCCI were searched for evidence related to young adults and adolescents.	Findings reveal that studies on youth access show that young people continue to obtain cigarettes from non-commercial sources (friends and family) and commercial sources (convenience stores).	Many of the results were not relevant to the research questions and outcomes of this review. Selected data has been used in the review.
Chaloupka *et al.* 1996 USA Cross-sectional +	N= nationally representative students in grade 8, 10 and 12.	Examines the effectiveness of several tobacco control policies in discouraging cigarette smoking among youth. Policies include limits on the availability of tobacco products to youth. Funded by the Centres for Disease Control and the Robert Wood Johnson Foundation.	Data was collected from the 1992–1994 Monitoring the Future campaign surveys of grade 8, 10, 12 students. Limits on the availability of tobacco products to youth were measured by several variables including: state, minimum legal purchase, age, etc.	Limits on youth access to tobacco products appear to have little impact on youth cigarette smoking, likely due to weak enforcement of the laws.	A well conducted study that disaggregated results based on gender and race. More information on confounders and missing data would have been useful.
Chaloupka *et al.* 1999 USA Cross-sectional +	N= 198, 359 nationally representative students in grade 8, 10 and 12. Authors do not provide ethnic breakdown, but state that sample was “nationally representative”	Examine differences in youth responsiveness to changes in price or tobacco control policies. Funded by the Centres for Disease Control and the Robert Wood Johnson Foundation.	Data was collected from the 1992–1994 Monitoring the Future campaign surveys of grade 8, 10, 12 students. Indexes examined gender, SES, race, cigarette consumption, etc.	Found significant differences in youth’s responsiveness to tobacco control initiatives by race. Smoking rates among white youth are significantly influenced by anti-tobacco activities and clean indoor air restrictions (p<0.05, p<0.10, respectively), whereas smoking rates among black youth are not. Smoking rates among black youth are significantly influenced by smoker protection laws and restrictions on youth access (ps<0.10), whereas smoking rates among whites are not.	A well conducted study that disaggregated results based on gender and race. More information on confounders and missing data would have been useful.
Difranza et al 2001 USA Cross-sectional +	N=2013 purchase attempts N=959 (1996) N=1054 (1997)	Evaluate merchant compliance with laws prohibiting the sale of tobacco to minors. Funded by the Massachusetts Tobacco Control Program.	Stratified cluster sampling was used to select outlets from which youth aged 13–17 years attempted to purchase tobacco.	Crude violation rates were 35% in 1996 and 17% for 1997 (p<0.001). Male clerks made more sales than female clerks (27% vs. 22%; p<0.05). Illegal sales were comparable for locked vending machines (19% of 47 attempts) and over the counter outlets (24% of 1075 attempts; p>0.05), but were more frequent in self service displays (37% of 75 attempts, p=0.01) vs. over the counter) and unlocked vending machines (64% of 58 attempts p<0.001 vs. over the counter). Sales occurred in 1.5% of the 1180 attempts when proof of age was requested, as compared with 64% of the 712 attempts when it was not (p<0.001). Sales occurred in 5% of 317 attempts when age was asked and in 30% of 1502 when it was not (p<0.001).	A well conducted study that discussed eligibility, sampling method and reliability of results. However, the study did not discuss reliability and validity of measurement methods and exposure, and did not discuss confounders.
DiFranza *et al* 1996 USA Cross-sectional +	N=480 cigarette purchase attempts. All of the tobacco merchants were located in 8 suburban and small urban communities. The over the counter vendors included convenience stores, pharmacies, liquor stores, and gasoline stations. All of the vending machines were located in restaurants. One boy and one girl aged 12, 13, 14, 15, 16, & 17 were recruited through acquaintances to attempt to purchase tobacco.	Evaluate the influence of age, gender, vending machine lockout devices and tobacco industry sponsored programmes (“It’s the Law” programmes) on underage youths’ ability to purchase tobacco. Funded by a grant from the Massachusetts Tobacco Control Programme.	12 young people made 480 attempts to purchase tobacco in Massachusetts from over the counter and vending machines with and without remote control lockout devices. Half the vendors were participating in “It’s the Law” programmes.	Youth were successful in 33% of their purchase attempts. Of the six opportunities to sell, 28% of the vendors never sold, 23% sold once, 16% sold twice, 9% sold three times, 13% sold four times, 6% sold five times, and 6% sold at every opportunity. Apparent age was a significant predictor of purchase success. Youth who appeared to be 16–17 years old were much more successful than youth who appeared to be 11–15 (OR=3.4, 95% CI= 2.0, 5.8, p=0.0001). Girls had a greater purchase success rate (OR= 1.49, 95% CI=1.01, 2.19, p<0.05). This persisted as a trend when apparent age was controlled in regression analysis (OR=1.59, 95% CI=0.94, 2.7, p=0.08). Boys (29%) and girls (28%) were equally likely to be asked for proof of age even though girls appeared older. Youth were much more successful purchasing from vending machines than from over the counter sources (OR= 3.0, 95% CI=1.9, 4.7, p=0.0001). In communities with no requirements for lockout devices, illegal sales were far more likely from vending machines than from over the counter sources (OR=5.9, 95% CI=3.3, 10.3, p=0.001). ‘It’s the Law’ programmes were not associated with a significant reduction in illegal sales when vending machine and over the counter sources were considered together (OR= 0.87, 95% CI=0.57, 1.35, p=0.5) or when they were considered separately.	A well conducted study that took many steps to reduce bias. However, confounders were not accounted for and eligibility criteria were not outlined.
Difranza *et al.* 1992 USA Cross-sectional -	N=156 tobacco merchants in Massachusetts	Examine the efficacy of the Tobacco Institutes “It’s the Law” program. Funder not mentioned.	5 underage youth, both male and female made “sham” purchase attempts from merchants participating in “It’s the Law” campaign.	Only 4.5% of 156 merchants were participating in “It’s the Law” program. 86% of merchants who were participating in the program were willing to illegally sell cigarettes to children, compared with 88% who were not participating.	There was a lack of information on sampling method, eligibility criteria, and the type of analysis conducted. No p-values were provided.
Fichtenberg *et al.* 2002 USA Systematic review +	N= 9 studies Inclusion criteria-studies must include compliance and prevalence data Interventions ranged in intensity from simple enforcement of laws to merchant and community education, to education combined with active enforcement via compliance testing, warnings, fines and suspension of tobacco selling licences.	To determine the effectiveness of laws restricting youth access to cigarettes on prevalence of smoking among teens. Funded by the National Cancer Institute.	Conducted a systematic review of studies that reported changes in smoking associated with the presence of restrictions on the ability of teens to purchase cigarettes. Calculated the correlation between merchant compliance levels with youth access laws and prevalence (30 day and regular) prevalence of youth smoking, and between changes in compliance and prevalence associated with youth access interventions. Conducted a random effects meta-analysis to determine the change in youth prevalence associated with youth access interventions from studies that included control communities.	There was no statistically significant relationship between merchant compliance and 30-day (r=0.116, p=0.486) or regular (r=0.017, p=0.926) teen smoking prevalence. There was no evidence that an increase in compliance with youth access restrictions was associated with a decrease in 30-day (r=0.294, p=0.237) or regular (r=0.274, p=0.287) prevalence. Although none of these correlations are statistically significant, their signs suggest a positive association between increased compliance and increased smoking prevalence. There was no significant difference in youth smoking in communities with youth access interventions compared with control communities: the pooled estimate of the mean difference in 30-day prevalence in the intervention group was −1.5% (95% CI - 6.0%, +2.9%)	A well conducted review. However it is not a Cochrane (which represents the benchmark for evidence-based medicine and reviews are conducted to extremely high standards).
Glanz *et al.* 2007 USA Cross-sectional ++	N=across eight years the number of stores surveyed ranged from: 448 in 1998 to 209 in 2003	Study examines the findings of annual Synar inspections to assess compliance with federal and state legislation to limit minors’ access to tobacco products in Hawaii. Study also reports on factors associated with selling tobacco to minors for the most recent year of inspections. Funded by Hawaii’s Department of Health’s Alcohol and Drug Abuse Division, Federal Substance Abuse Prevention and Treatment Block Grant and the	Annual random unannounced inspections were conducted by minors over an 8 year period (1996–2003). Stores were randomly selected from a list of stores that sell tobacco products in Hawaii.	There was a decrease in the percent of successful purchases made over the period from 1996 to 2003 (44.5% vs. 6.2%). Based on multivariate analysis, only 2 variables were associated with whether a successful purchase attempt was made in 2003: whether the minors’ age (OR = 0.030, 95% CI =0.002, 0.426) or identification (OR = 0.001, 95% CI = 0.001, 0.020) was requested.	A very well conducted study that accounted for confounders, had a high participation rate, and dealt with missing data.
		Hawaii Tobacco Control Settlement Fund.			
Lantz *et al.* 2000 USA Review (narrative synthesis) +	N= not clear how many articles were reviewed (However there are 142 references in the reference list).	To provide a comprehensive review of interventions and policies aimed at reducing youth cigarette smoking in the US, including strategies that have undergone evaluation and emerging innovations that have not yet been accessed for efficiency. Funded from Mr. Ted Klein, president of Ted Klein and Co., a New York City public relations firm.	Medline literature searches, books, reports, electronic list servers, and interviews with tobacco control advocates. Intervention and policy approaches were categorised into seven categories (school based, community interventions, mass media/public education, advertising restrictions, youth access restrictions, taxes and direct restrictions on smoking.	Youth smoking prevention control efforts have had mixed results. However, this review suggests a number of prevention strategies that are promising, especially if conducted in a coordinated way to take advantage of potential synergies across interventions. Several types of strategies warrant additional attention and evaluation including aggressive media campaigns.	A well conducted review, however, studies were limited to the US. Furthermore, it is not a Cochrane review which is the benchmark for evidence-based medicine and reviews.
Landrine *et al.* 1996 USA Cross-sectional +	N=2,567 purchase attempts from 72 stores. Thirty-six children (18 girls, 18 boys) were recruited to participate in the study. There were 12 children in each of the three age groups (10-, 14-, and 16-year-olds) and 12 in each of the three ethnic groups (whites, Latinos, and African Americans)	Examined the role of asking age/ID in cigarette sales to minors and explored the possible demographic correlates of asking such questions. Funded by Cigarette and Tobacco Surtax Fund of the State of California through the University of Calif. Tobacco Related Disease Research Program.	36 minors, representing equal numbers of girls, boys, whites, blacks and Latinos of 10, 14, and 16 year olds each attempted to purchase cigarettes once from each of the 72 stores. The frequency of asking the children their age and/or for ID was analyzed along with the role of these questions in subsequent sales.	The data revealed that requesting age/ID was rare (occurring 17% of the time) despite the laws in California. If clerks asked children their age, sales were significantly less likely (x^2^=36.3, p<0.001). When age was asked, minors were refused cigarettes 95.8% of the time. Similarly, if clerks requested ID, sales were significantly less likely (x^2^=16.8, p<0.001). When ID was requested, minors were refused cigarettes 99% of the time. Requesting ID was more strongly associated with decreased sales than asking age.	Good reliability and validity, however, the study dates were not clear, confounders were not addressed and missing data was mentioned but not accounted for.
Levinson *et al.* 2002 USA Non-randomised controlled trial +	N=1083 purchase attempts	To estimate the effect on cigarette sales rates when minors present ID Funded by State Tobacco Education and Prevention Partnership, Colorado Dept. of Health and Environment	Controlled experiment in which minors attempting to purchase cigarettes either carried a valid ID (documenting that they were minors) or carried no ID< and were instructed to show their ID or admit having no ID if the clerk requested proof of age.	When clerks requested ID, sales were more than 6 times as frequent if minors presented ID than if they did not (12.2% vs. 2.0%, RR = 6.2, p<0.0001).	A well conducted study that adequately addressed concealment, treatment and control groups and comparison of results across sites.
Levy and Friend. 2002 USA Review (narrative synthesis) +	N= 23 studies nationally representative sample	To review empirical studies of youth access policies to better understand the components of successful and unsuccessful interventions and their impact on youth smoking rates. The purpose of this review is to formulate future policies and create a framework for additional research	Interventions: Included enforcement efforts to reduce access by minors at stores, vending machines and social sources. The relationship between youth access policies and smoking rates is inconsistent. The researchers also found that in many cases the intervention had only short-term results.	The researchers found that a successful policy that reduces retail sales usually has a multi-component approach that includes severe enforcement and penalties, as well as community education and mobilization.	A well conducted review that adequately addressed the significance of combining community, mobilization and enforcement to tackle smoking among youth.
Price 1998 New Zealand Cross-sectional -	N=980 stores were visited for controlled purchase operations (CPO’s) between 1996–1997	Reports on the initiative-increased enforcement of section 30(1) which prohibits the sale of tobacco products to persons under the age of 18. Funder not mentioned.	Ministry of Health coordinated a programme of CPO’s using under age volunteers to identify merchants illegally selling tobacco products to minors.	Between Sept 1996 and Jun 1997, 693 CPO’s were conducted, and 67 (9.7%) resulted in the sale of tobacco to minors. Between July and Dec 1997, a further 287 CPO’s were conducted and 17 (5.9%) resulted in sales. Therefore a total of 980 CPO’s were conducted, with 84 (6.8) resulting in sales. Of the 49 merchants prosecuted to date (December 1997), 41 were convicted.	No information on the type of analysis and no info on sampling frame. There was a general lack of information.
Ross *et al.* 2006 USA Cross-sectional +	N=16, 558 youth in grades 9–12.	Examine the differential effects of cigarette prices, clean indoor air laws, youth access laws and other socio-economic factors on smoking uptake among US high school students. The study also examines whether those at the final stages of uptake are more price responsive than those at the beginning stage. Funder not mentioned.	Youth in grade 9–12 completed the “study of smoking and tobacco use among young people” survey. Questions examined actual smoking behaviour, risk of uptake among non-smokers, and numerous variables examining SES, ethnicity, gender and age.	Compliance with youth access laws reduced the probability of being in a higher stage of smoking uptake (p<0.05). The finding that the impact of compliance is larger for those who are in later stages supports the hypothesis that social sources of cigarettes are more important in the earlier stages of smoking uptake.	A well conducted study, however, there was no baseline or comparison and no information on missing data (readers are told the data is missing but we are not told how this impacts the results).
Stead *et al.* 2005 UK Cochrane Review (narrative synthesis) ++	N=34 studies (14=had data from a control group for at least one outcome) Review included controlled trials and uncontrolled studies with pre and post intervention assessment of interventions to change merchants’ behaviour.	1) Does the intervention with merchants, by education, active enforcement of laws, or combinations of strategies lead to decreased sales to minors? Is there evidence that any of the strategies is superior to the others? 2) Do reduced sales of tobacco to minors lead to a decrease in their self reported ease of access? 3.) Do reduced sales of tobacco to minors reduce the prevalence of tobacco use? Sources of support: NHS Research and Development Programme UK, Department of Primary Health Care, University of Oxford UK.	Assess the effects of interventions to reduce underage access to tobacco by deterring shopkeepers from making illegal sales. Interventions: The review considered education, law enforcement, community mobilization, or combinations of strategies that aimed to deter merchants from selling tobacco to minors.	Giving merchant’s information was less effective in reducing illegal sales than active enforcement or multi-component educational strategies, or both. No strategy achieved complete, sustained compliance. In three controlled trials, there was little effect of intervention on youth perceptions of access or prevalence of smoking.	The Cochrane reviews represent the benchmark for evidence-based medicine, and reviews are conducted to extremely high standards.
Sundh *et al.* 2006 Sweden Cross-sectional +	N= 3150 test purchases in three regions of Sweden. Purchase attempts were made in supermarkets, food stores, after-hours supermarkets, newsagents and gas stations. 28 phone interviews with individuals in the tobacco prevention field (regional and local levels).	Study the possible changes in adolescents’ opportunities for purchasing tobacco during the period 1996–2005. The study also investigated regional differences in adolescents’ opportunities for purchasing tobacco, and elucidated the efforts by authorities to affect the compliance with the minimum age law of 17. Funded by the National Institute of Public Health in Sweden.	In 1996, 1999, 2002, and 2005, 3150 test purchases of tobacco were conducted in controlled forms by 48 adolescents in three regions of Sweden. In addition, 28 structured phone interviews were conducted with key people in tobacco prevention work.	In 1996, 84% of test purchases in shops with a voluntary age limit resulted in successful purchases. A significant decline was observed in 2005, 8 years after the minimum age tobacco law was introduced, with 48% of test purchases resulting in successful purchases (p <0.001). Results showed differences between the three regions (p values ranging from 0.001 to 0.01) in compliance and in activities connected with the minimum age tobacco law.	This study was well conducted but lacked information on eligibility criteria, and was missing data (i.e. why specific communities were not involved in the study). Interview data/results were also lacking (rich data was not provided; all responses were categorized into three categories).
Sundh et al 2005 Sweden Cross-sectional +	N=20,130 (1996) N=21,492 (2000) Youth were 13, 15 and 17 years old.	The purpose of this study was to increase understanding of the prerequisites for tobacco prevention. The situations before and after the introduction of a minimum age law were compared with respect to opportunities for adolescents to buy tobacco, and to attitudes towards the law. Funded by the National Institute of Public Health in Sweden.	Data was collected in 1996 and 2000 with a questionnaire examining tobacco, alcohol, drugs, health, family finances etc. Specific questions asked youth for their attitudes towards the minimum age law	Findings revealed that the proportion of boys and girls in year 7 who said that they had bought tobacco during the previous month had decreased significantly from 11.5% to 7.8% and from 11.6% to 6.9%, respectively (both p<0.0001). (p<0.0001) between 1996 and 2000, whereas the corresponding figures for older adolescents remained unchanged. Restricting the analysis to smokers, the proportion of girls who bought tobacco in shops decreased in all ages groups (Year 7: 93.8% to 74.1%; Year 9: 94.3% to 84.8%; Year 2 of upper secondary school: 96.4% to 90.7%, p<0.001). Corresponding figures for boys showed a statistically significant decrease only among year 9 students (92.8% to 87.6%, p<0.05).	A well conducted study that discussed the type of analysis conducted and eligibility. However, there was a lack of information on missing data, confounders and reliability.
Sundh et al 2004 Sweden Cross-sectional +	N=1,500 purchase attempts N=750 purchase attempts made before the law N=750 after the law	Purpose of the study is to compare the possibility of adolescents purchasing tobacco before and after the introduction of a minimum age law of 18 years, and to examine the factors that characterize the situations in which adolescents may or may not purchase tobacco. Funded by the National Institute of Public Health in Sweden.	Under controlled conditions adolescents of varying ages carried out test purchases of tobacco.	In 1996, 91% of purchase attempts were successful, whereas in 1999, 82% of purchase attempts were successful (p<0.001). Requests for age or ID substantially decreased the likelihood of successful purchase.	A well conducted study that included a baseline survey. However, participants who carried out test purchase attempts were legal (18 years old), and simply looked young. This could raise issues of reliability and validity.
Tangirala *et al.* 2006 USA Cross-sectional +	N= 5096 retail outlets in the state of Indiana including 1367 (26.82%) chain stores, 3729 (73.18%) independently owned stores. A total of 326 primary tobacco outlets were also identified via a database.	Determine whether inspections are effective as a means of increasing merchant compliance in restricting sales to persons under the age of 18 years, especially among merchants who have violated the law in the past. This project is supported by the Master Tobacco Settlement fund through the Indiana Tobacco Prevention and Cessation Agency-administered through the Alcohol & Tobacco Commission and the Indiana Prevention Resource Centre.	Secondary data analysis was performed on inspection date from 2001–2003. The investigative team identified tobacco retail outlets with more than one inspection within the last 19 month time frame.	The percentage of violations at Inspection 2 was significantly lower than the percentage of violations at Inspection 1 (25.9% vs. 32.3%, p<0.05), indicating that retail outlet inspections are associated with increased sales restrictions to youth.	Study was well conducted and outlined eligibility criteria. Study also does a good job of outlining limitations. However, it failed to account for confounders, and missing data.
Tutt *et al.* 2000 Australia Cross-sectional -	N= 133 vendors (1994) N= 126 (1995) N=124 (1996) N= 44 (1996/97) N= 51 (1997/98) N=47 (1998/99) *Sample of merchants surveyed has been in decline as a result of store closures. Merchants to be tested : all those located within a 3km radius of four high schools located across the research area plus the nearest main shopping centre.	Examine retail compliance with prohibition of sales to minors. Proportion of youth smoking was also examined. Funder not mentioned.	Retail compliance with prohibition of sales to minors was monitored through a series of undercover compliance surveys between 1993 and 1999. Compliance rates were affected by a campaign aimed at increasing merchant awareness of their obligations under the new law and well publicised prosecutions. Intervention: education and awareness of Public Health Act (prohibition of selling tobacco to minors). Active enforcement of law in 1995.	In 1996 seven successful prosecutions occurred across the study area, with most resulting in $1000 penalties and extensive publicity. Since then only three merchants have been successfully prosecuted, 2 in 1997 and 1 in 1999. Non-compliance in surveys dropped from 30.8% (1994) to 8.1% in May 1996. The overall proportion of 12–17 year olds reporting at least monthly smoking dropped from 25.9% in 1993, to 22.7% in 1996, and to 17.1% in 1999. Greatest reductions were in youth who smoked “less than 1 a day”, or “1–5 a day” (x2=18.4, p=0.182).	Confounders mentioned but not accounted for. Study outlined eligibility criteria and response rates. However, changes in the types and intensity of the intervention likely changed compliance checks.

## **Appendix A:** Smoking uptake and young people search strategies.

- young person* or young people or young adult* or young individual*
- under 18* or underage* or under eighteen*
- boy or boys or girl or girls
- child* or adolescen* or kid or kids or youth* or youngster* or minor or minors or teen* or juvenile* or student* or pupil or pupils
- smoking or antismoking or anti-smoking or smoker or smokers or tobacco
- cigar* or bidi or bidis or beedi or beedis or kretek or handroll* or hand roll* or nicotine
- (sale or sales or sell or selling or sold or supply or supplies or supplied or supply*) within 3 (tobacco or cigar* or bidi or bidis or beedi or beedis or kretek or handroll* or hand roll* or nicotine)
- (purchase* or retail*) within 3 (tobacco or cigar* or bidi or bidis or beedi or beedis or kretek or handroll* or hand roll* or nicotine)
- (buy or buys or buying or bought) within 3 (tobacco or cigar* or bidi or bidis or beedi or beedis or kretek or handroll* or hand roll* or nicotine)
- (vend or vends or vending) within 3 (tobacco or cigar* or bidi or bidis or beedi or beedis or kretek or handroll* or hand roll* or nicotine)
- (shop or shops or shopping or shopped) within 3 (tobacco or cigar* or bidi or bidis or beedi or beedis or kretek or handroll* or hand roll* or nicotine)
- (store or stores or supermarket*) within 3 (tobacco or cigar* or bidi or bidis or beedi or beedis or kretek or handroll* or hand roll* or nicotine) tobacconist*
- (prevent* or regulat* or control* or restrict* or prohibit* or ban* or limit* or illegal or law or legislat*or policy or policies) within 3 (smoke or smoking or tobacco or cigar* or bidi or bidis or beedi or beedis or kretek or handroll* or hand roll* or nicotine)
- limit to (english language and yr="1990–2007")

## **Appendix B.** Excluded Studies

**Table:**

*Paper*	*Reason for exclusion*
Altman, D.G.; Wheelis, A.Y.; McFarlane, M.; Lee, H.; Fortman, S.P. The relationship between tobacco access and use among adolescents: a four community study. *Soc. Sci. Med.***1999**, *48*, 759–775.	Covered in Cochrane Review. Community-based intervention: Monterey County, CA
Altman, D.G.; Rasenick-Dous, L.; Foster, V.; Tye, J.B. Sustained Effects of an Educational Program to Reduce Sales of Cigarettes to Minors. *Amer. J. Public Health***1991**, *81*, 891–893.	Covered in Cochrane Review. Community-based intervention: Santa Clara County, CA.
Banerjee, S.C.; Green, K. Analysis Versus Production: Adolescents Cognitive and Attitudinal Responses to Antismoking Interventions. *J. Commun.***2006**, *56*, 773–794.	Not an intervention.
Chapman, S.; King, M. Effects of publicity and a warning letter on illegal cigarette sales to minors. *Aust. J. Public Health***1994**, *18*, 39–42.	Covered in Cochrane Review.
Chen, V.; Foster, J.L. The long-term effect of local policies to restrict retail sale of tobacco to youth. *Nicotine Tob. Res.***2006**, *8*, 371–377.	Community-based intervention: 14 communities in Minnesota.
Cheng, T.O. Peer, Parental, and Commercial Influences on Cigarette Smoking among Chinese Youth. *J. Natl. Med. Assn.***2004**, *96*, 691–692.	Not an intervention. Commentary.
Cummings, K.M.; Hyland, A.; Saunders-Martin, T.; Perla, J.; Coppola, P.R.; Pechacek, T.F. Evaluation of an enforcement program to reduce tobacco sales to minors. *Amer. J. Public Health***1998**, *88*, 932–936.	Covered in Cochrane Review.
Cummings, K.M.; Saunders-Martin, T.; Clarke, H.; Perla, J. Monitoring vendor compliance with tobacco sales laws: Payment vs. no payment approaches. *Amer. J. Public Health***1996**, *86*, 750–751.	Not relevant to research question.
Curran, J.J., Jr. Preventing youth access to tobacco products in Maryland. *Maryland Med. J.***1995**, *44*, 793–195.	Not an intervention
Dovell, R.A.; Mowat, D.L.; Dorland, J.; Lam, M. Changes among retailers selling cigarettes to minors. *Can. J. Public Health***1996**, *87*, 66–68.	Covered in Cochrane Review. Community-based intervention: local intervention
Feighery, E. The effects of coming education and enforcement to reduce tobacco sales to minors: a study of four northern California communities. *J. Amer. Med. Assn.***1991**, *266*, 3168–3171.	Covered in Cochrane Review. Community-based intervention: 4 cities in Solano County, California
Forster, J.L.; Murray, D.M.; Wolfson, M.; Blaine, T.M.; Wagenaar, A.C.; Hennrikus, D.J. The effects of community policies to reduce youth access to tobacco. *Amer. J. Public Health***1998**, *88*, 1193–1198.	Covered in Cochrane Review.
Forster, J.L.; Hourigan, M.E.; Kelder, S. Locking devices on cigarette vending machines: Evaluation of a city ordinance. *Amer. J. Public Health***1992**, *81*, 1217–1219.	Covered in Cochrane Review. Community-based intervention: St. Paul, MN
Gemson, D.H.; Moats, H.L.; Watkins, B.X.; Ganz, M.L.; Robinson, S.; Healton, E. Laying down the law: Reducing illegal tobacco sales to minors in central Harlem. *Amer. J. Public Health***1998**, *88*, 936–939.	Covered in Cochrane Review,
Goldstein, A.O.; Sobel, R.A.; Martin, J.D.; Crocker, S.D.; Malek, S.H. How does North Carolina law enforcement limit youth access to tobacco products? *N. C. Med. J.***1998**, *58*, 90–94.	No outcomes of interest.
Jason, L.A.; Ji, P.Y.; Anes, M.D.; Birkhead, S.H. Active enforcement of cigarettes control laws in the prevention of cigarette sales to minors. *J. Amer. Med. Assn.***1991**, *266*, 3159–3161.	Covered in Cochrane Review. Community-based intervention: Santa Clara, CA.
Jason, L.A.; Berk, M.; Schnopp-Wyatt, D.L.; Talbot, B. Effects of enforcement of youth access laws on smoking prevalence. *Amer. J. Commun. Psychol.***1999**, *27*, 143–160.	Covered in Cochrane Review. Community-based intervention: Woodridge, IL.
Jason, L.A.; Billows, W.D.; Schnopp-Wyatt, D.L.; King, C. Long-term findings from Woodridge in reducing illegal cigarette sales to older minors. *Eval. Health Prof.***1996**, *19*, 3–13.	Covered in Cochrane Review. Community-based intervention: Woodridge, IL
Jason, L.A.; Katz, R.; Vavra, J.; Schnopp-Wyatt, D.L.; Talbot, B. Long-term follow-up of youth access to tobacco laws’ impact on smoking prevalence. *J. Hum. Behav. Soc. Environ.***1999**, *2*, 1–13.	Covered in Cochrane Review. Community-based intervention: Woodridge, IL.
Jason, L.; Billows, W.; Schnopp-Wyatt, D.; King, C. Reducing the illegal sales of cigarettes to minors: Analysis of alternative enforcement schedules. *J. Appl. Behav. Anal.***1996**, *29*, 333–344.	Covered in Cochrane Review. Community-based intervention: Chicago, IL.
Junck, E.; Humphries, J.; Rissel, C. Reducing tobacco sales to minors in Manly: 10 months follow-up. *Health Promot. J. Aust***1997**, *7*, 29–34.	Covered in Cochrane Review. Community-based intervention: Manly, a suburb in Sydney, Australia.
Keay, D.K.; Woodruff, S.I.; Wildey, M.B.; Kenney, E.M. Effect of retailer intervention on cigarette sales to minors in San Diego County, California. *Tob. Control***1993**, *2*, 145–151.	Covered in Cochrane Review. Community-based intervention: San Diego County, CA
Krevor, B.S.; Liebermn, A.; Gerlach, K. Application of consumer protection authority in preventing tobacco sales to minors. *Tobacco Control***2002**, *11*, 109–111.	Not an intervention. No outcomes of interest. Special communication, descriptive study.
Krevor, B.; Capitman, J.A.; Oblak, L.; Cannon, J.B.; Ruwe, M. Preventing illegal tobacco and alcohol sales to minors through electronic age-verification devices: a field effectiveness study. *J. Public Health Policy***2003**, *24*, 251–268.	No outcomes of interest. Not relevant to research question.
Perla, J.P. Effects of increase retailer compliance rates on youth smoking behaviours and access to cigarettes. Ph.D. thesis, State University of New York at Buffalo: Buffalo, NY, USA, 1998, pp. 1–163.	Community-based intervention: 13 suburban communities in Erie County, NY.
Powell, L.M.; Chaloupka, F.J. Parents, public policy, and youth smoking. *J. Policy Anal. Manag.***2005**, *24*, 93–112.	No relevant outcomes. Emphasis on parental influences on smoking behaviour.
Powell, L.M.; Taurus, J.A.; Ross, H. The importance of peer effects, cigarette prices, and tobacco control policies on youth smoking behaviour. *J. Health Economics***2005**, *24*, 950–968.	Tobacco control policies that were examined included local level policies. Furthermore, the paper was not focuses on prevention-participants were smokers. Key focus of paper was impact of peers on smoking.
Powell, L.M.; Chaloupka, F.J. Parents, public policy, and youth smoking. *J. Public Policy Anal. Manag.***2005**, *24*, 93–112.	Key focus of paper was impact of parents on smoking. Lack of information on access restrictions. Access restrictions examined went beyond those within the scope of this review (i.e. packaging).
Rigotti, N.A.; DiFranza, J.R.; Chang, Y.; Tisdale, T.; Kemp, B.; Singer, D.E. The effect of enforcing tobacco-sales laws on adolescents’ access to tobacco and smoking behaviour. *N. Engl. J. Med.***1997**, *337*, 1044–1051.	Covered in Cochrane Review. Community-based intervention: 5 Massachusetts communities.
Siegel, M.; Biener, L.; Rigotti, N. The effects of local tobacco sales laws on adolescent smoking initiation. *Prev. Med.***1999**, *29*, 334–342.	Community-based intervention: local communities in Massachusetts
Skretny, M.T.; Cummings, K.M.; Sciandra, R.; Marshall, J. An Intervention to reduce the sale of cigarettes to minors in New York State. *N. Y. State. J. Med.***1990**, *92*, 54–55.	Covered in Cochrane Review.
Staff, M.; Bennett, C.M.; Angel, P. Is restricting tobacco sales the answer to adolescent smoking? *Prev. Med.***2003**, *37*, 529–533.	Covered in Cochrane Review. Community based intervention: 11 northern Sydney metropolitan public secondary schools.
Thomson, C.C.; Gokhale, M.; Biener, L.; Siegel, M.B.; Rigotti, N.A. Statewide evaluation of youth access ordinances in Practice: Effects of the implementation of a community-level regulation in Massachusettes. *J. Public Health Manag. Pract.***2004**, *10*, 481–489.	Community-based intervention: communities in Massachusetts.
Widley, M.B.; Woodruff, S.; Agro, A.; Keay, K.; Kenney, E.M.; Conway, T.L. Sustained effects of educating retailers to reduce cigarettes sales to minors. *Public Health Rep.***1995**, *110*, 625–629.	Covered in Cochrane Review. Community-based interventions: San Diego County, California.
